# BUILDING INCLUSIVE COMMUNITIES FOR PEOPLE WITH DEMENTIA: TAKING THE LEAD FROM LIVED EXPERIENCE

**DOI:** 10.1093/geroni/igae098.1498

**Published:** 2024-12-31

**Authors:** Alison Phinney, Lynn Jackson, Ania Landy, Mariko Sakamoto

**Affiliations:** University of British Columbia, Vancouver, British Columbia, Canada; University of British Columbia, Vancouver, British Columbia, Canada; University of British Columbia, Vancouver, British Columbia, Canada; University of Victoria, Victoria, British Columbia, Canada

## Abstract

Social citizenship is increasingly adopted in academic circles as a theoretical perspective foregrounding the rights of people with dementia to full participation in their community. However, what this means in practice is poorly understood. Using methods of participatory action research and developmental evaluation, our team conducted a four-year study to learn what social citizenship means from the perspective of people with lived experience, and to put that understanding into action through community programs and services. In phase one, an action group of people with dementia identified stigma as their main concern, and together with the research team co-developed an online toolkit to raise awareness and “flip stigma on its ear”. In phase two, the toolkit was shared with community stakeholders who provided formative feedback, and the team conducted implementation sessions to explore the toolkit’s impact for community programs and services who wanted to be more dementia inclusive. Analysis of interviews with stakeholders and program leaders and field notes from the feedback and implementation sessions identified key themes about how the toolkit has been received and its impact across varying community contexts, e.g. libraries, churches, community centres, and adult day programs: (1) the power of hearing real voices; (2) recognizing stigma; (3) gaining confidence for change; (4) spreading the word; and (5) working with (not for) people with dementia. Overall, these groups found that taking the lead from people with lived experience has been pivotal in helping them take first meaningful steps toward dementia inclusion.

